# Honoring Drs. McCosh and Bull

**Published:** 1909-04

**Authors:** 


### Honoring Drs. McCosh and Bull.

UNDER this heading *The New York Tribune* editorially in its edition of March 21, 1909, considers the appropriateness of the proposed memorials to these two distinguished deceased surgeons. Already something more than $100,000 has been subscribed in the case of Dr. McCosh and it is probably that the Presbyterian Hospital will be removed to the site selected by Dr. McCosh.

No doubt, quite as large a sum will be subscribed in case of Dr. Bull. We let the *Tribune* speak for itself.

HONORING DRS. M'COSH AND BULL.

So distinguished were the services rendered to the community by two surgeons whose deaths have occurred within the last few months that there is peculiar propriety in the movements to recognise their work with permanent memorials. Dr. McCosh having been closely identified with the Presbyterian Hospital, that institution will naturally be the site of whatever token of appreciation is erected in his honor. Dr. Bull's intimate association with the College of Physicians and Surgeons (the medical department of Columbia University) lends equal fitness to the proposition to establish some monument to his memory in the well known 59th street school.

One of the suggestions concerning the form which the memorial to Dr. Bull should take is that provision be made for a laboratory for surgical research. The plan is warmly approved by *The New York Medical Journal,* which prefers an endowment to a statue, because, in the opinion of that periodical, "the beneficent work of a hospital, a college or a laboratory is destined to be more lasting and by its name to perpetuate a man's memory more forcibly."

The project certainly constitutes a happy expression of sentiment—the prime object to be kept in view—and yet aims to aid in an extremely practical manner the profession to which Dr. Bull was so devoted a servant. It would promote both the benevolent and scientific agencies of which his life was a striking embodiment. Realising, as he did, the limitations of surgical knowledge, Dr. Bull himself might well have wished that any tribute to him should take such shape.

